# Quantification of Three-Dimensional Cell-Mediated Collagen Remodeling Using Graph Theory

**DOI:** 10.1371/journal.pone.0012783

**Published:** 2010-09-30

**Authors:** Cemal Cagatay Bilgin, Amanda W. Lund, Ali Can, George E. Plopper, Bülent Yener

**Affiliations:** 1 Computer Science Department, Rensselaer Polytechnic Institute, Troy, New York, United States of America; 2 Biology Department, Rensselaer Polytechnic Institute, Troy, New York, United States of America; 3 General Electric Global Research Center, Niskayuna, New York, United States of America; Dalhousie University, Canada

## Abstract

**Background:**

Cell cooperation is a critical event during tissue development. We present the first precise metrics to quantify the interaction between mesenchymal stem cells (MSCs) and extra cellular matrix (ECM). In particular, we describe cooperative collagen alignment process with respect to the spatio-temporal organization and function of mesenchymal stem cells in three dimensions.

**Methodology/Principal Findings:**

We defined two precise metrics: *Collagen Alignment Index* and *Cell Dissatisfaction Level*, for quantitatively tracking type I collagen and fibrillogenesis remodeling by mesenchymal stem cells over time. Computation of these metrics was based on graph theory and vector calculus. The cells and their three dimensional type I collagen microenvironment were modeled by three dimensional cell-graphs and collagen fiber organization was calculated from gradient vectors. With the enhancement of mesenchymal stem cell differentiation, acceleration through different phases was quantitatively demonstrated. The phases were clustered in a statistically significant manner based on collagen organization, with late phases of remodeling by untreated cells clustering strongly with early phases of remodeling by differentiating cells. The experiments were repeated three times to conclude that the metrics could successfully identify critical phases of collagen remodeling that were dependent upon cooperativity within the cell population.

**Conclusions/Significance:**

Definition of early metrics that are able to predict long-term functionality by linking engineered tissue structure to function is an important step toward optimizing biomaterials for the purposes of regenerative medicine.

## Introduction

Natural and engineered tissues are a collection of cells arranged within a structural scaffold of extracellular matrix (ECM) proteins that provide biochemical and mechanical cues to direct function. This function is therefore dependent upon the spatio-temporal resolution of matrix proteins, cells and signaling molecules. 3D extracellular matrices in vitro and in vivo affect the structural, mechanical and biochemical make-up of the cellular micro-environment and are crucial for a bidirectional interplay to exist between the cell and tissue during development [1], [2]. For this reason, they are often used in functional tissue engineering. However, the mechanisms that define the 3D interface between cells and the surrounding matrix and how cells cooperate to affect that matrix have yet to be determined.

The bidirectional remodeling of the ECM during morphogenetic events is required for structure development and function. For example, during blood vessel formation endothelial cells initiate neovascularization by unfolding soluble fibronectin (FN) and depositing a pericellular network of fibrils that support their intracellular cytoskeleton [3]. This fibrillogenesis is necessary for tubule formation and its inhibition prevents proper lumen formation and polarization. Additionally, both engineered and natural alignment of collagen fibers define cell morphology, organization and function in engineered tissues [4]. These changes in the architecture of the ECM are sensed by cellular adhesions and propagated through the cytoskeleton and signaling cascades to affect nuclear organization, chromatin structure and gene expression [5]. Mesenchymal stem cells (MSC) use the ECM to direct and maintain their self renewal and differentiation potential in vivo. Recapitulation of this control in vitro is the “gold standard” for MSC tissue engineers.

Using multiphoton optical microscopy and second harmonic generation (SHG) we can non-invasively image type I collagen fibers in 3D. SHG microscopy visualizes the nano-periodic, non-centrosymmetric structure of type I collagen fibers with the use of high intensity light and does not require labeling of the collagen network [6]–[8]. Tracking of SHG signal allows for the analysis of type I collagen fibrillogenesis, represented by fibril alignment and consolidation over time [9], [10]. When encapsulated within a 3D type I collagen microenvironment, cells initially contract to compact the matrix and subsequently remodel the disordered, and entangled fibrillar network created following gelation [11]–[13]. Quantitatively understanding the interplay between cell population and the dynamics of the local microenvironment is critical to developing mechanistic hypotheses governing the role of the cell/matrix interface in tissue homeostasis, development and repair. To create a quantitative method to achieve this aim we have created a tool based upon the hypothesis that cells within developing tissues must cooperate (through coordinated push and pull forces) to align and remodel their local microenvironment.

3D confocal imaging techniques have been a powerful tool for cell biologists and engineers providing spatial information regarding the location of specific structures within cells and tissues. Critical to the throughput and effectiveness of imaging studies, however, is the development of methods for quantitative and automated analysis of multi-spectral images over time. The translation of these informative but inherently qualitative images into quantitative metrics is necessary to guide tissue engineering design and the rigorous testing of mechanistic hypotheses in 3D.

Developing methods to efficiently move from biological hypothesis to successful in vivo application, requires rigorous methods of analyzing the spatio-temporal function of tissues. Modeling functional, temporal and spatial networks presents itself as a powerful tool for the testing of these hypotheses and improving the throughput of biomaterial optimization. Models inherently provide quantitative analysis that provides feedback loops to inform engineering design [14]–[16]. From the single cell level up to the level of developing tissue and organization dynamics, developing models that quantitatively capture the principles guiding each tier of decision making is an emerging field that stands to accelerate tissue engineering efforts.

In [17], [18] the authors work has clearly shown the importance of the ECM on stem cell fate decisions but their work has focused on directing stem cell fate decisions where tools that allows us to measure in 3D over time “how” and “when” stem cells interact with their environment to alter their own fate are still in need.

To this point, efforts at quantifying the microstructure of type I collagen fibers in native tissue as well as engineered constructs have based their results on 2D images including scanning [19] and transmission [20], [21] electron microscopy, histological samples [22] and synthetic data [23]. While electron microscopy provides high resolution images they are less precise than confocal fluorescence images as they represent metal replicas of static tissues. Current methods of extracting quantitative metrics from these types of images include localized vector analysis [21], Fourier transform [24], mean intercept length and line fraction deviation methods [23]. These methods provide bulk material properties that describe fiber alignment, concentration, anisotropy but do not quantify the dynamic changes in collagen microstructure and organization with time. Additionally, current technologies examine collagen alignment in isolation, and thus fail to capture the relationship between changing structure and cellular organization. The coordination of cells and the surrounding matrix defines tissue function and therefore it is the dynamic interaction between these two components that provides critical mechanisms in tissue development.

In this work we have developed a quantitative method of analyzing the bidirectional cell/matrix interface. We provided a method of probing 3D structure in its native form over time and linking matrix dynamics with cellular organization and function using graph theory. In our previous work we have developed a modeling and mining methodology based on graph theory (called the cell-graphs) to study tissue organization and its corresponding functional state in the context of automated cancer diagnosis for brain [25], breast [26], and bone [27] tissues. Most recently, we used cell-graph approach to study MSC organization [28] over time.

This study extends our previous work to 3D cellular environment to define and compute two novel metrics, namely Collagen Alignment Index and Cell Dissatisfaction Level. These metrics could track and quantify type I collagen remodeling and fibrillogenesis with respect to mesenchymal stem cell organization over time.

We compare our techniques developed to physics based mechanical models [29] in which each pair of neighboring cells (identified using a Delaunay triangulation) is attached by a spring. Two main differences are (1) cell-graph based establishment of neighborhoods and pairwise relationships (i.e., edges), (2) replacing the “springs” with weighted edges where weights are calculated directly from images of collagen fibers.

Our results are verified on multiple experiments and provide the first quantitative support to the hypothesis that continuity between extracellular and intracellular environments is required for stem cell fate determination.

## Results

### A novel metric “Collagen Alignment Index” is defined and computed to quantify the intimate cooperative relationship that exists between a stem cell and its developing microenvironment

**Figure 1:**
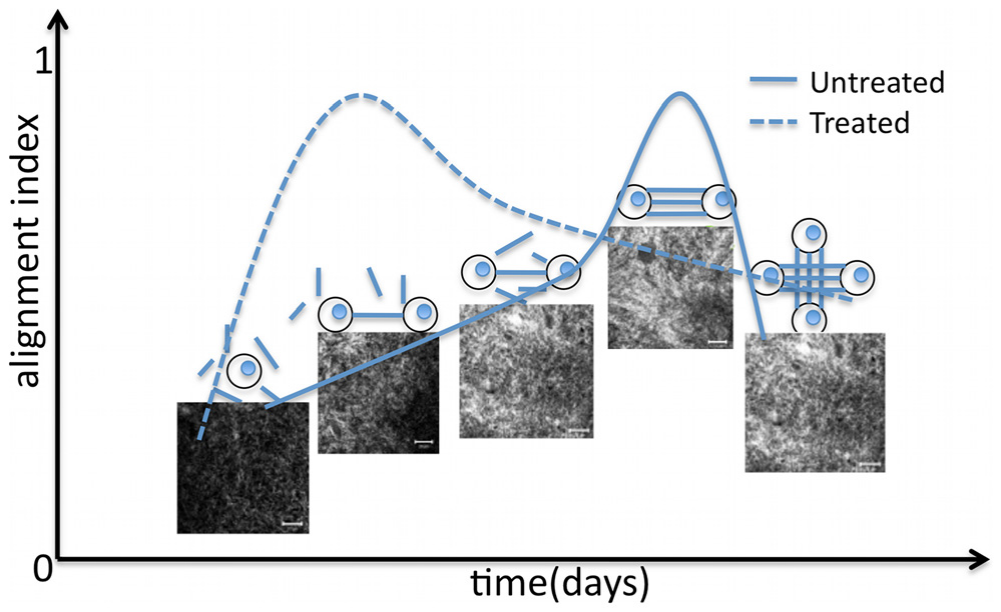
This schematic describes a conceptual model for MSC mediated collagen alignment during early phases of hydrogel compaction in 3D culture developed from results presented here. MSC interact and engage their type I collagen microenvironment to organize it over time. This process occurs via three distinct and phases, I, II and III. Untreated gels progress through these phases as cells first begin to interact with their surrounding collagen independently and then engage with cells in their immediate environment to cooperatively pull, and align the collagen matrix. They peak here at Collagen Alignment Index CAI approaching to 1. As the cells continue to interact they loose this directionality and begin to cooperate in more complex ways with cells surrounding them resulting in a reentry into early clusters of collagen organization (IIb and Ib). Intermediate phases can be indicative of different interactions as depicted above. When induced to differentiate through treatment with the MEK inhibitor PD98059 (treated), MSC accelerate through these early phases of remodeling to efficiently direct their cooperativity and slowly reorganize. Plots are indicative of the cooperativity metric Collagen Alignment Index, solid line (untreated) and dashed line (treated). Inset images represent second harmonic generation images of the type I collagen matrix indicative of each stage of remodeling and schematics are given to represent the hypothesized cooperation occurring between cells.

In this work, we present a model for quantifying the dynamics of cell/ECM interaction based upon the hypothesis that cells within developing tissues must cooperate (through coordinated push and pull forces) to align and remodel their local microenvironment. We present two key metrics that quantify the intimate cooperative relationship that exists between a stem cell and its developing microenvironment. From this work we can define a model of mesenchymal stem cell (MSC) remodeling over time, Figure 1, in which MSC remodel the disorganized type I collagen in their local environment over time through cooperative pulling and alignment of the collagen fibers. Furthermore, alignment is accelerated when MSC are induced to differentiate through treatment with the MEK inhibitor, PD98059. In this work we introduce a novel metric, the *Collagen Alignment Index* (CAI) which peaks at 90, representing complete alignment between two cells, after 2 days as shown in Figure 1.

MSC embedded in 3D type I collagen gels were used as a model of stem cell osteogenic differentiation and tissue remodeling [30]. In this model, the cells were seeded at 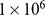 cells per mL in 2mg/mL type I collagen. Over the course of three days, the gels underwent extensive compaction accompanied by remodeling of the type I collagen microstructure. After seven days, the cells began expressing osteogenic genes and eventually deposit a calcified matrix, hallmarks of in vitro osteogenic differentiation [30]. Furthermore, previous work found that the addition of the MEK inhibitor, PD98059, accelerates this differentiation event and induces significant changes in observed collagen remodeling.

CAI (Collagen Alignment Index) measures pulling between cells and alignment of collagen fibers as two cells cooperate to create order in their random and disorganized microenvironment. The computation of this metric requires (i) obtaining the relative density and position of fibrillar type I collagen in ECM, and (ii) modeling of cell-to-cell interactions.

**Figure 2:**
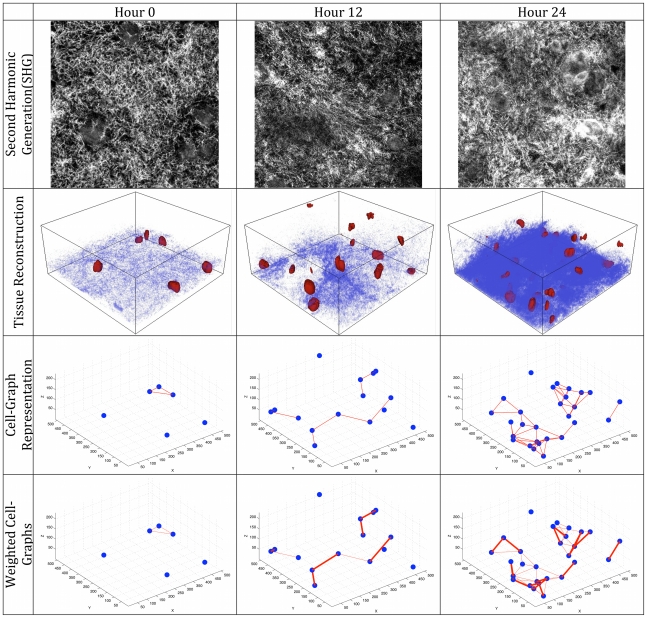
General methodology for quantifying collagen alignment and the structural organization of the tissue. First row: one optical slice of a 3D second harmonic generation multiphoton confocal image (Scale bar 20
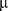
m). Corresponding confocal images of the cell nuclei were segmented using the Otsu Thresholding algorithm. Connected pixels were found in this segmented image and each connected component was labeled as an individual cell nuclei. Using these segmented confocal images of nuclei, we reconstructed and visualized the tissue on top of the collagen in 3D (Second row). For each nucleus, the center of mass was found and assigned as the x,y,z coordinates of that nucleus. Using the nuclei locations, cell-graphs that capture the spatial relationship between the nuclei were constructed and visualized (Third row). The collagen alignment around every edge of the graph was quantified and the Collagen Alignment Index that measures the quality of the alignment is assigned to each edge. Cell-graph edges that have an alignment greater than a given threshold (in this case 0.6) were drawn thicker to highlight areas of enhanced remodeling in 3D (Fourth row).

**Figure 3:**
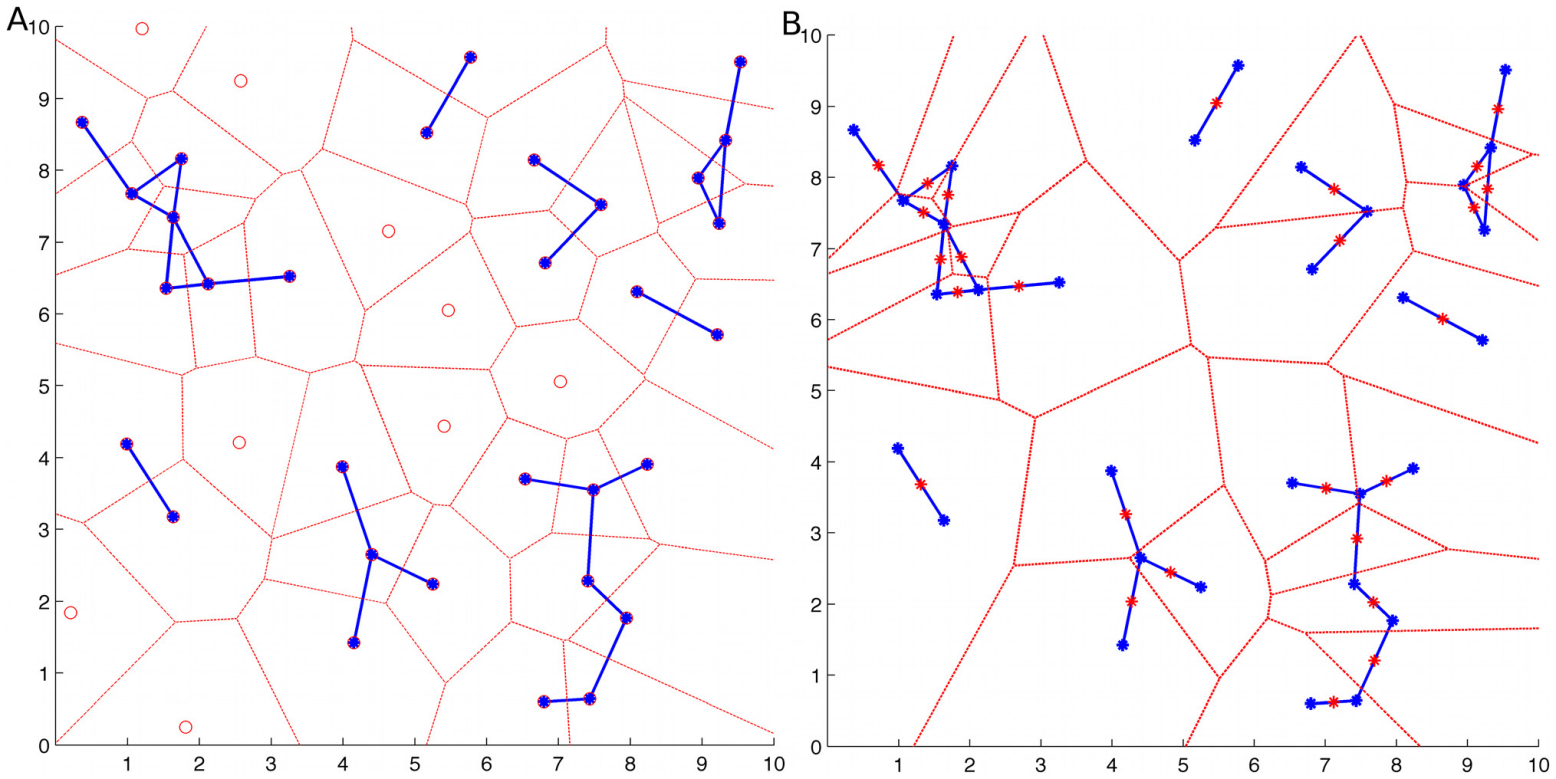
Edge-based and Node-based Voronoi partitioning. Two different Voronoi construction techniques were used in this work, done both from a cell-graph node perspective and from a cell-graph edge perspective. In Figure 3(a), a sample Voronoi diagram using the cell coordinates as the seed points is shown where blue (node with edges) and red (node without edges) circles are the cell nodes. Blue lines correspond to cell graph edges while dashed red lines correspond to the Voronoi compartments. In this original state of the Voronoi diagram, each cell-graph edge was shared by two separate Voronoi compartments. In Figure 3(b), to capture the information between cells the method was altered to set the center of the edges as the seed points. This construction ensures that each cell-graph edge is encapsulated in only one Voronoi compartment. To capture the information between cells the method was altered to set the center of the edge as the seed point. This construction ensures that each cell-graph edge is encapsulated in only one Voronoi compartment. These compartments were projected onto the corresponding SHG image to assign each pixel of type I collagen signal to a given graph node (A) or edge (B).

**Figure 4:**
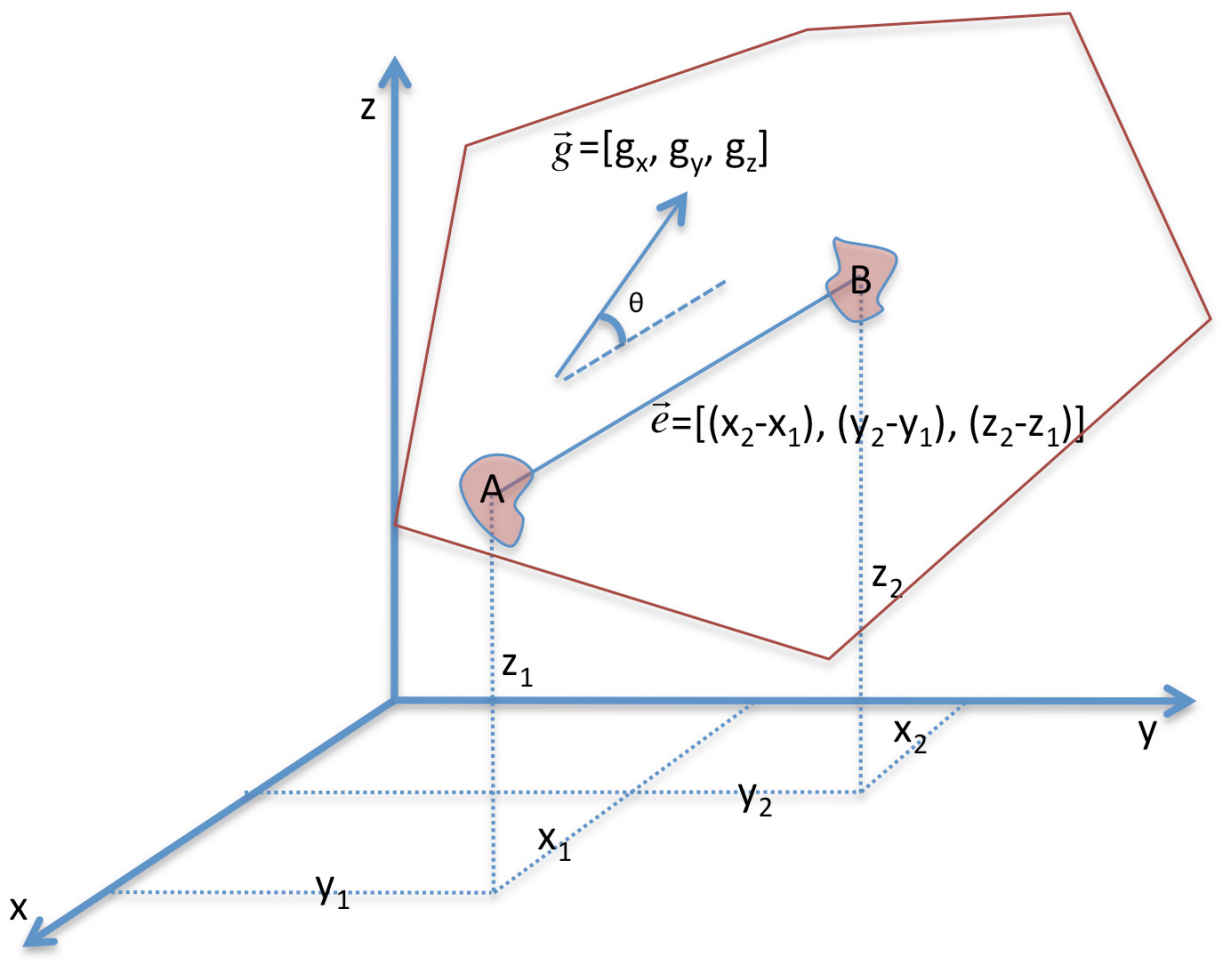
Gradient vector analysis for quantification of type I collagen structure. Given a segmented image and voronoi construction we can quantify type I collagen alignment with respect to either the graph edge or node. Two cells, A, B are depicted in 3D space. The coordinates of these cells are given as 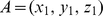, 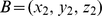. The cell-graph edge was therefore represented as the vector 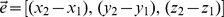. The red line gives the boundaries of the edge-based Voronoi diagram. For each pixel in this compartment the gradient vector that measures the direction of the maximum intensity change is calculated and the angle 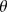 between the gradient vector and the cell-graph edge was calculated. Using the distribution of these angles, or the sum of all the gradient vectors we assessed direction and magnitude of collagen organization in 3D space.

**Figure 5:**
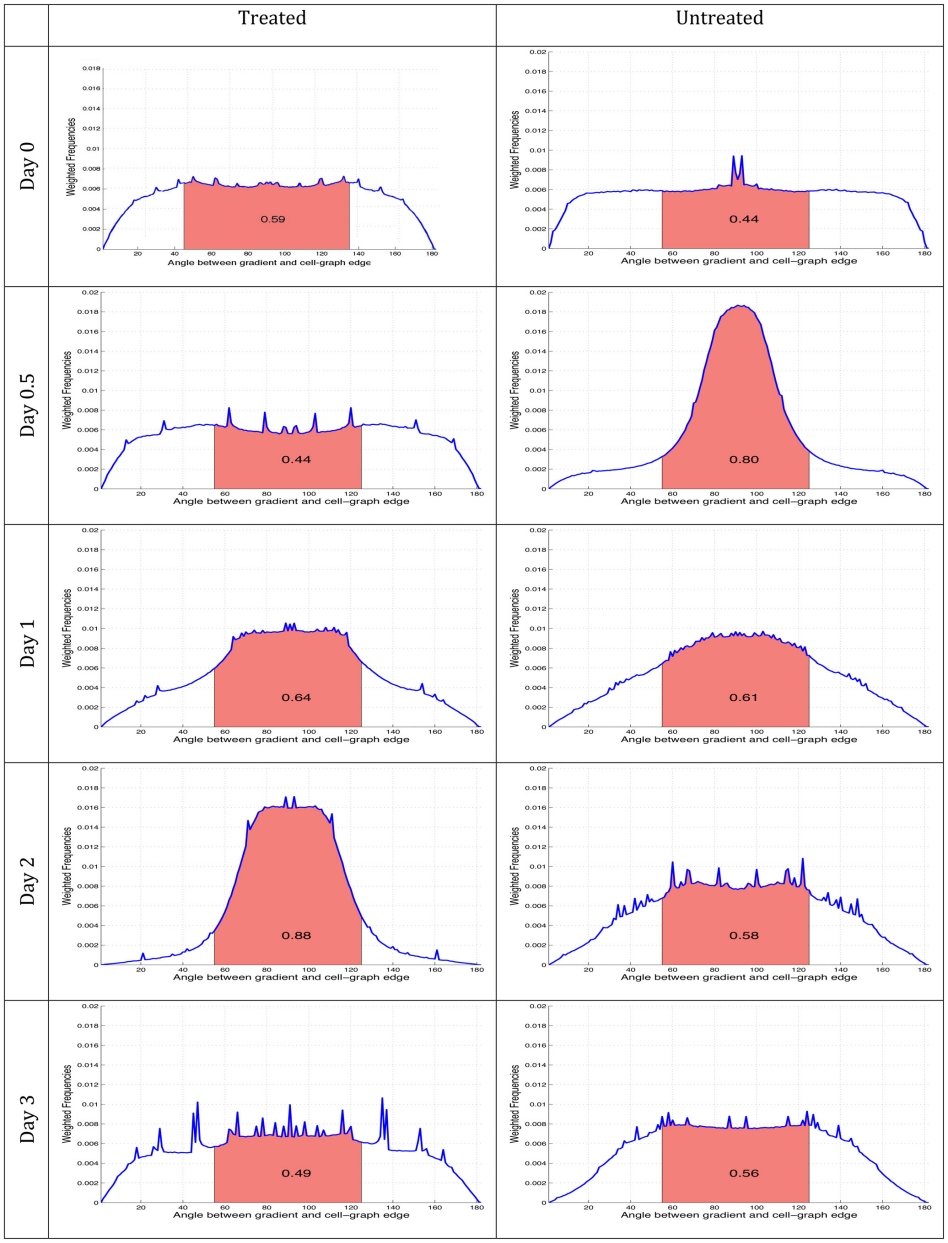
Quantification of MSC cooperativity during type I collagen fibrillogenesis. The distribution of the angles between the gradient vectors and cell-graph edges are plotted for untreated and treated (shown as PD) samples at each time point imaged. Histograms plot the frequency of each angle found within the corresponding set of images weighted according to the gradient magnitudes. The area under the curve between 60 and 120 degrees (red shaded area) is assigned as the Collagen Alignment Index for that sample and shown in the middle of the curve. This metric attains its maximum value at day 2 in the untreated tissue sample whereas in the treated tissue samples this value takes its maximum value as early as hour 12. Using these distributions, the samples were clustered into three groups by k-means algorithm. The first and the last tissue samples of both the untreated and treated tissues grouped in one cluster, day 2 of untreated and hour 12 of treated example were grouped in the second cluster and all the other samples clustered as a third. This analysis was performed on distinct biological samples, n = 3.

For the former, we collected 3D multiphoton confocal images of cell position (estimated by location of fluorescent nuclei) for 0, 0.5, 1, 2 and 3 days after gel formation and the relative density and position of fibrillar type I collagen (by second harmonic generation (SHG) microscopy), Figure 2. For the latter, the image stacks of the nuclei were segmented and reconstructed in 3D. We constructed *cell-graphs* to connect cells (nodes) in 3D space based upon 3D Euclidean distances. Links (edges) were added between cells based upon a distance threshold set at 55 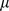
m, approximated by two rounded cell diameters, Figure 2. The 3D space was partitioned using Voronoi diagrams to ensure that each cell-graph node, Figure 3(a), and each cell-graph edge resided in a unique 3D compartment Figure 3(b). The gradient vector of each pixel was then computed within each compartment. In general, the gradient vector points in the direction of the maximum change in the intensity value and the magnitude of the gradient vector gives the value of that change. Given the gradient vectors and the cell-graph edges, the angle between the two, denoted by 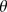, was found as in Figure 4 and CAI metric was calculated as histograms of the angles 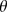 , Figure 5. Therefore, as the gradient points in the direction of the maximum intensity change, a 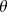 of 90 degrees is representative of perfect alignment: collagen fibers running perpendicular to a cell graph edge running between two adjacent cells.

Our results obtained over three independent experiments, indicate that CAI metric can (i) track type I collagen remodeling and fibrillogenesis with respect to mesenchymal stem cell organization over time, and can (ii) extract critical phases of early cell-mediated collagen remodeling and demonstrated acceleration through these phases with the induction of differentiation.

### Differentiating MSC accelerate their progression through three distinct phases of type I collagen fibrillogenesis

The normalized histograms i.e., probability density functions (pdfs) of the 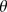 values defined above suggested a relationship between the treated group at hour 12 and the untreated group at day 2, Figure 5. We used the Kolmogorov-Smirnov test (KS test)to verify that the pdfs were coming from the same distribution.

Since the KS test is more accurate near the center of distributions, the left and the right tails of the distributions were not considered. After limiting the pdfs within the range of [60,120] (recall that perfect alignment is quantified by a 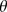 value of 90), the KS test accepted the hypothesis that these two distributions originated from the same distribution with a p-value of 0.1070. All other pdf pairs were also compared but the KS test did not point out any other significant inter/intra relationships between tissue samples over three independent experiments.

Singular value decomposition (SVD) and k-means statistical tests demonstrated that MSC in type I collagen gels progress through three stages during early compaction events (I, II and III), Table 1. Just after encapsulation MSC have yet to exert an organizational force on their type I collagen matrix. Phase I represented this disorganization through a broad and random distribution of 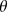. As the MSC interacted with their microenvironment they compacted the hydrogel and organized the collagen fibers, Figure 5 (insets). Quantification of 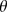 demonstrated that this organization was first achieved through alignment of type I collagen fibers parallel to the cell-graph edge. Distribution of 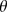 narrowed to reach a peak at 90 indicative of a net gradient vector perpendicular to the edge. The distribution of 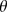 re-broadened and increased complexity was observed in the types of angles that were found with respect to the edges Figure 5. Untreated MSC progress from Phase I to Phase II within 12 hours and Phase III was reached by day two. Time 0 and day 3 clustered together indicating statistically similar distributions of 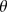 and were therefore both classified as Phase I.

**Table 1:** Clustering of treated and untreated samples.

	Tissue Type	
Time points (days)	untreated	treated
0	I	I
0.5	II	III
1	II	II
2	III	II
3	I	I

Singular value decomposition (SVD) and k-means statistical tests demonstrated that MSC in type I collagen gels progress through three stages during early compaction events I, II and III.

Interaction with the extracellular environment drives MSC differentiation events. PD980598, a MEK inhibitor, was used to enhance the osteogenic potential of MSC in type I collagen hydrogels, [30]. Over the same time course SHG images were taken and reconstructed, cell-graphs were built, and gradient vector analysis was completed. Statistical comparisons between the pdfs of 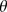 for untreated and treated (PD98059) gels demonstrated that each gel set moved through statistically similar phases. The rate with which the treated gels progressed through these phases, however, was altered. Differentiating cells quickly progressed to Phase III (as defined by the untreated gels) and then slowly reorganized to reach the final Phase I. The treated gels came out of Phase III much slower than untreated, moving through Phase II after Phase III and finally to Phase I, as shown in Figure 1. The collagen gels used in tissue engineering are magnitudes less stiff than even the softest tissues in the body. This requires intense remodeling by stem cells, particularly in cases for tissue engineered products but is also very relevant in wound healing, so that they can replicate that tissue without diminishing the integrity of the tissue. For this reason the alignment of fibers in a parallel fashion and then the complex overlay of groups of such fibers would be required to begin to replicate the properties of in vivo tissue. Collagen compaction results in better material properties and therefore compaction of collagen based products presents a natural way to replicate the complexity of the fibrous in vivo structure. From our analysis, we saw that type I collagen gels progress through the following stages: (i) Phase 1: Initial organization directly around the cell (ii) Cells beginning to make contacts with nearest neighbors resulting in the parallel fibers between cells and finally (iii) Phase 3 where as cells become more compact in space their interactions with neighbors become more complex. The CAI (Collagen Alignment Indenx) metric is a quantitation of these values and lets us put any given tissue within a range of tissue development over time.

### A novel feature “Cell Dissatisfaction Level” quantifies the microenvironment dynamics and predicts homeostasis

Type I collagen is organized during hydrogel compaction by interactions between neighboring cells as well as directly by individual cells. While the CAI metric captures impact of cell-to-cell interactions on the microenvironment (computed over cell-graph edges), here we introduce a new metric computed for an individual cell to complement it.

Formally, the gradient vector points in the direction of the maximum change in the intensity value and the magnitude of the gradient vector gives the value of that change. The gradient vector represents the net direction and magnitude of the force exerted by the cell onto the collagen in its immediate 3D neighborhood Figure 4. Gradient vector analysis was performed on a node by node (cell by cell) basis. Thus, for each tissue sample, 3D space is partitioned (using Voronoi partition) into compartments that included exactly one unique node (cell) Figure 3(a). Similar to CAI metric, for each pixel within each compartment, the gradient vector was calculated.

In contrast with CAI metric where we built histograms, all the gradient vectors within a compartment were summed to calculate a net gradient vector to be assigned to the cell in the particular Voronoi cell. Thus, at the end of this step each cell in the tissue is assigned a direction indicating “net” pulling of ECM. Increased local organization in the area of the node resulted in a larger value for the net gradient vector quantifying directional interaction between the cell and its microenvironment.

**Figure 6:**
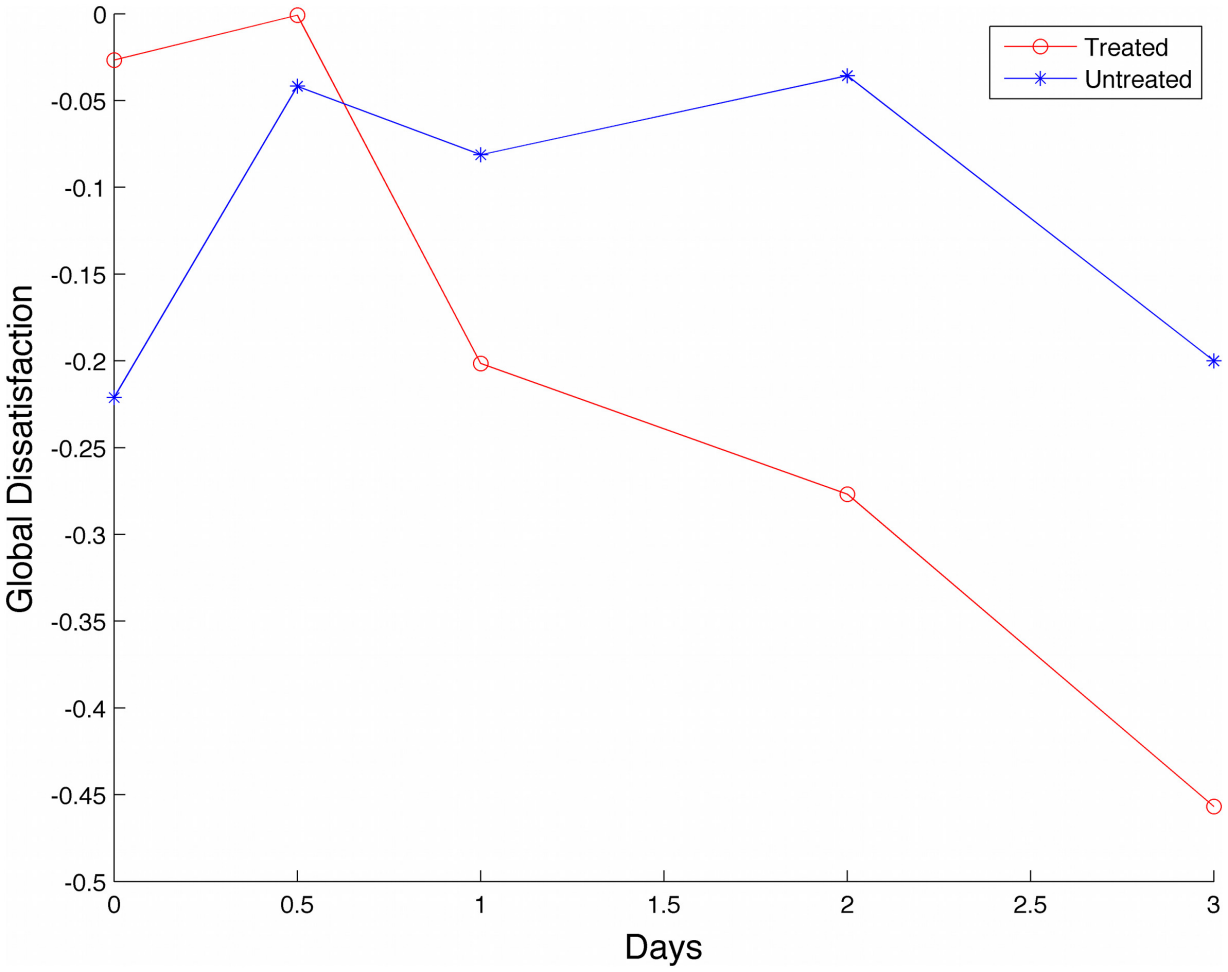
MSC in treated type I collagen gels exhibit accelerated satisfaction. Node-based Voronoi diagrams were constructed as in Figure 3(a) and gradient vectors calculated for each pixel within the compartment. Using node-based voronoi construction, collagen alignment around an individual cell was captured. The gradient vectors were summed and assigned as the net force to the nucleus in the respective Voronoi compartment. To find the magnitude of these individual forces, the net gradient vector was projected onto the cell-graph edges using the minimum norm projection method. The magnitudes of these projections on each cell-graph edge were assigned as the weight of that edge. Using these weights the overall tissue structure and collagen formation in the tissue were represented by a weighted graph, Figure 2. The weights of each edge are summed and the resulting sum is the Global Dissatisfaction Level (GDL) of the sample, normalized to image size. The global dissatisfaction of the treated samples decreases more rapidly when compared to untreated controls.

The net gradient vector of a cell (node) is then written as a linear combination of the cell-graph edge vectors incident to node which produces a system of linear equations. The solution of this system assigns each cell-graph edge a weight called *Cell Dissatisfaction Level* (CDL) to quantify the dynamic remodeling ‘forces’ exerted by individual cells on their local microenvironment Figure 6.

Tissue satisfaction is reached upon homeostasis when the forces exerted by cells in a local area are balanced. The global Cell Dissatisfaction Level of the tissue was found by summing the weights projected onto each cell graph edge. In a perfect stability of the microenvironment, edge weights should cancel out to minimize the unbalanced pull forces.

We observed that MSC in type I collagen gels slowly progress from a dissatisfied state towards a more satisfied state (decrease in dissatisfaction) over time as fibrillogenesis and reorganization occurs. When treated to differentiate MSC accelerate their progression towards a more ‘satisfied’ state, Figure 6. By filtering out the weights of the cell-graphs that are less than a certain threshold, local microenvironment where fibrillogenesis and reorganization has reached a certain degree can be found and visualized as depicted in last row of Figure 2.

## Discussion

The main aim of this work is to provide precise metrics to quantify the MSC-ECM interactions that govern collagen alignment, remodeling and differentiation. We introduce two such metrics: Collagen Alignment Index (CAI) and Cell Dissatisfaction Level (CDL). Both metrics are computed over 3D images. While the former directly quantifies physical forces applied to collagen fibers in the ECM between a pair of cells, the latter determines when the dynamic microenvironment will reach to a state of equilibrium: homeostasis.

We used human mesenchymal stem cells (MSC) embedded in 3D type I collagen gels as a model of stem cell osteogenic differentiation and tissue remodeling [30]. Using this model we traced the dynamics of cell/ECM interactions during differentiation and remodeling events. A MEK inhibitor, PD980598, was used to enhance the osteogenic potential of MSC in type I collagen hydrogels and over the same time course SHG images were taken. These images were processed, the cell-graphs were formed and gradient vector analysis is completed. A weighted, normalized histogram of the angle between a possible communication link and the collagen fibrils was calculated. Statistical analysis of these histograms for untreated and treated (PD98059) were performed. Our analysis extracted critical phases of early cell-mediated collagen remodeling and demonstrated acceleration through these phases with the induction of differentiation. We further see that each gel set moved through statistically similar phases. The rate with which the treated gels progressed through these phases however, was altered.

We established links between cells to model cell-to-cell interaction in a graph theoretical sense using the cell-graph methodology. The cell-graph methodology represents each cell with a vertex (node) and establishes a link between a pair of nodes if there is a biologically sound hypothesis that the pair communicates with each other through chemical signals, ECM etc. An edge can be established deterministically (if the cell membranes are in physical contact) or probabilistically (as a function of the distance between the nodes).

Using cell-graphs, the structural organization of tissue samples are captured. Cell-graphs previously used to distinguish between different functional states in 2D histopathological slides of brain, breast and bone tissues with up to 98 and 84 and 90 percent accuracy [25]–[27]. Cell-Graphs are the generalizations of (Voronoi) Delaunay graphs to arbitrary edge functions and have two main advantages over Delaunay triangulations [31]. First, Delaunay triangulations are planar graphs and therefore do not allow the crossing of edges. Second, the result of Delaunay triangulation is a single connected graph. There is no evidence to justify these two assumptions. Cell-graphs remedy these two shortcomings of Delaunay graphs and therefore model the spatial distribution of the cells and temporal evolution of the tissue more precisely. Nevertheless Voronoi construction has its advantages as well. On a tissue image, the Voronoi diagram partitions the image into convex polygons such that each polygon contains exactly one seed point (also referred as generating point) and every point in a given polygon is closer to its seed point than to any other seed point in the tissue. Voronoi diagrams have been used to estimate the boundaries of cells in a tissue when membrane staining is not present [32], [33].

A careful analysis of the probability density functions of each sample in Figure 2 suggests that there is a relationship between the second time point of treated and fourth time point of untreated sample. The implication of this observation is that treated samples mature faster than the untreated ones and a high collagen alignment exists during the progression of the tissue. The Kolmogorov-Smirnov test is used to decide if two datasets (i.e., the second time point of treated and fourth time point of untreated sample) are coming from the same distribution. We used the KS-test since it makes no assumption about the distribution of data and it is a non-parametric statistical test.

The generality of the KS-test comes at the cost of being less accurate than others if the specific data meets all the assumptions of the other test in hand. For example, Student's t-test may be more sensitive if the data follows a standart normal distribution. Since we did not want to make an assumption of that sort, KS-test proved to be the more accurate one. In general, there are three limitations of the KS-test. First, the distributions are expected to be continuous. Second, the test is more sensitive near the center of distribution than at the tails and third the distribution must be fully specified. The distribution of 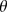 values runs from 0 to 180. To meet the assumptions of the KS-test, angles between 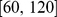 were used as inputs. A good collagen alignment results in angles close to 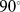 as discussed before. Therefore clipping the histogram does not disturb the calculation of the Collagen Alignment Index (CAI) and is consistent with our previous analysis.

Fibrillar collagens (types I, II, III, V, XI) form a basic triple helical structure that can self-assemble into highly organized fibrils. Given this structure, these collagens provide significant tensile strength and a structural frame for tissues, including bone, skin, blood vessels and intestine. Type I collagen is by far the most prevalent collagen within the bone matrix and assembles spontaneously in the extracellular space in staggered fibrils. Mutations within the collagen I gene, that result in the improper assembly of fibrils, lead to osteogenesis imperfecta, characterized by extreme bone fragility.

Collagen fibers in bone are organized at 90 degree angles to each other to create a plywood-like material, lending tensile strength to the tissue. The self-assembly of staggered collagen fibers yields a consistent 60nm repeating unit that is visible in electron microscopy images of mature collagen fibers. It is this non-centrosymmetric property of the collagen fibers that results in strong second harmonic signal.

To capture both the nuclei information and the collagen information 40× magnification is used during imaging from microscopy. At this magnification segmentation of the collagen organization is not possible. We therefore used the gradient information of each image to assess the collagen fibril formation. Voronoi diagrams of tissue samples were constructed to find the boundaries of cells. For each pixel in each Voronoi compartment, the gradient vector is calculated. The gradient of an image and the edges in that image are closely related. In our case, the gradients give information about the collagen fibrils in the SHG channel. For each pixel in each Voronoi compartment, we sum the gradient magnitude and assign it as the *net pull* by the collagen to the cell located in that compartment.

Once these forces are projected onto the cell-graph edges, the tissue sample will be represented as directed weighted graphs where the weights are the norms of each projection vectors. These directed, weighted cell-graphs capture both the spatial information of the cells and the force applied by the collagen to cells. The directed weighted cell-graphs can then be used as an in silico model of the spatial distribution of the cells and the collagen alignment between cells.

The exact distribution of the collagen alignment angle is dependent on the topology of the graph. Our cell-graph construction technique has one parameter, namely the link threshold, that gives the maximum allowed distance for two cells to communicate with each other. The higher this edge threshold is, the more cell-graph edges are introduced in the construction. The edge lengths cannot be smaller than the length of the shorter axis of cell membrane. We performed a parametric search and repeated our experiments within the range of 10 to 180 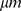 to find that a specific edge length between 55, 60 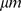 gives the observed faster maturation.

Our conclusion is as follows: Using the methods developed through this work we have demonstrated metrics that describe, track and quantify both local and global mesenchymal stem cell-mediated type I collagen fibrillogenesis and remodelling. These metrics are used to assess the dynamic bidirectional cell/matrix interface in their native form over time. As a result in silico representations of matrix dynamics linked with cellular organization and function are obtained.

Our methods represent the type I collagen microenvironment by cell-graphs that capture the structural properties of the tissues. Tissue samples are partitioned into meaningful subspaces to capture the possible communication link and its neighborhood between two cells. Type I collagen organization is quantified in this region and assigned as edge weights between a pair of cells to further model the collagen organization as well as structural properties of the tissues.

Modeling the interaction between cells and their local microenvironment provides a powerful quantitative and analytic tool for assessing function of engineered tissues as well as for understanding the basic science of tissue development, homeostasis and repair. We present here metrics to define and quantify this process which will allow for the testing of hypotheses within dynamic, 3D engineered tissue constructs.

## Materials and Methods

**Figure 7:**
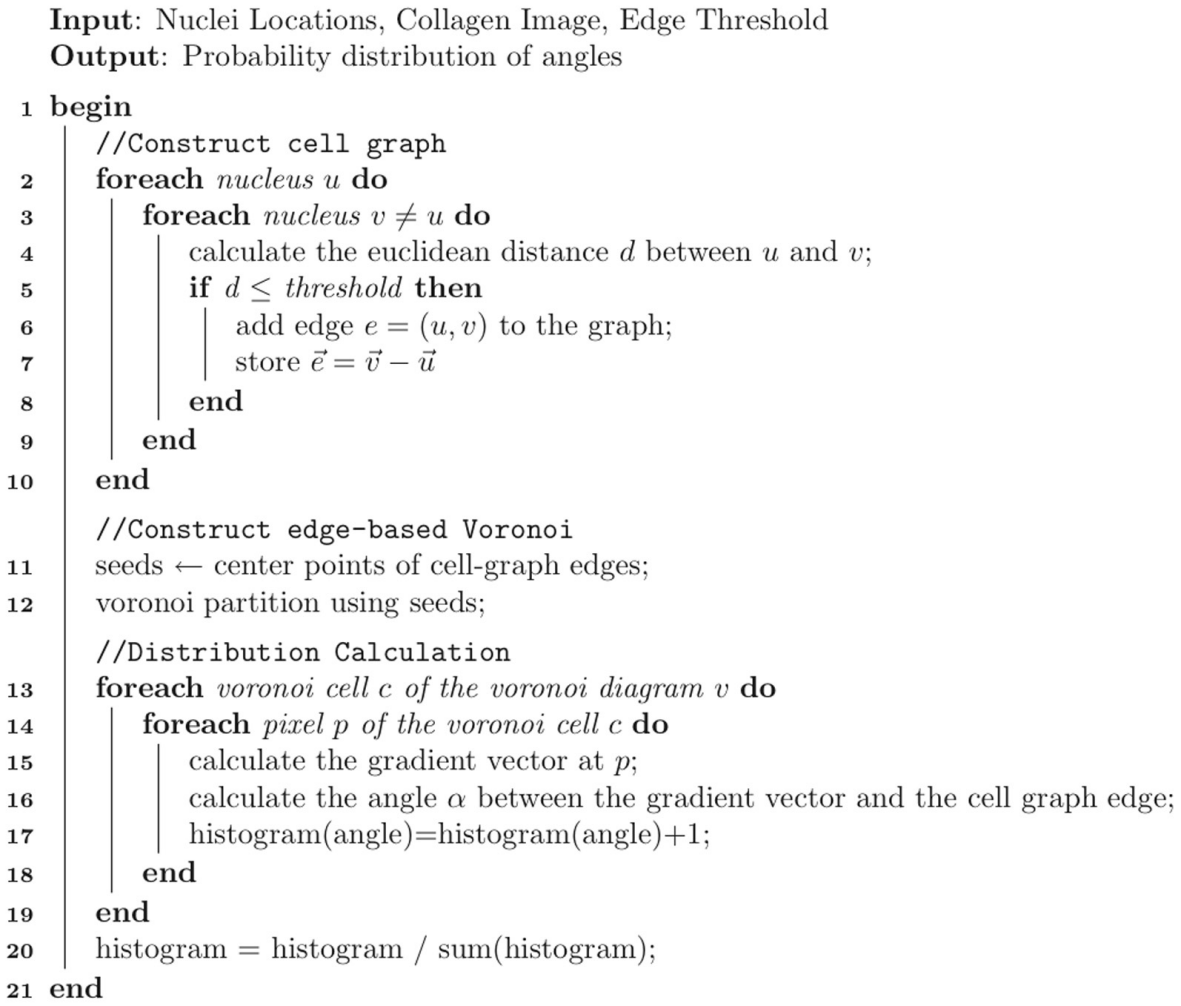
Outline of the algorithm to compute Collagen Alignment Index (CAI). After segmenting the nuclei, cell-graph are built capturing the spatial distribution. Voronoi diagrams are built and the gradient vectors in each Voronoi compartment are calculated. A distribution of the angles between the cell-graph edges and the gradient vectors is calculated to find the CAI metric.

Our methodology as depicted in Figure 7, has following steps: (1) growing hMSC, (2) preparation of the three-dimensional collagen I gels, (3) imaging and image analysis of the 3D gels, (4) building cell-graphs to model cell-to-cell interactions, (5) calculation of the gradient vectors, and (6) computing the metrics. The first four steps are based on our previous work [28] and necessary for the computation of the Collagen Alignment Index and Cell Dissatisfaction Level. In the following subsections we explain each of the steps in more detail.

### Cell Culture

Cryopreserved hMSC (Lonza) were grown according to manufacturer's instructions. hMSC were cultured in Dulbecco's Modification of Eagle's Medium 1× (DMEM) supplemented with 10% fetal bovine serum (FBS) and fungizone/penicillin/streptomycin (FPS) [10,000 units/mL]. Medium was changed every three days and cultures were incubated at 37

C in a humidified atmosphere containing 95% air and 5% CO2. Cells were detached using trypsin-EDTA and passaged into fresh culture flasks upon reaching confluence. hMSC were used between passages 6 and 8. In preparation for incorporation into 3D constructs, cells were washed with phosphate saline buffer Ph 7.4 (PBS), detached with trypsin-EDTA, collected, and counted. ERK activity was controlled through the inhibition of its upstream activator MEK using PD98059 dissolved in DMSO (Calbiochem). Cells were pre-incubated with PD98059 at a concentration of 50 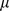
M for 15 min and then encapsulated within a collagen hydrogel for 3D culture as described below. Constructs were grown in PD98059 supplemented medium for the given time points. The final DMSO concentration never exceeded 0.1% and the same amount of the DMSO vehicle was added to control samples. All reagents were purchased from Fisher Scientific unless otherwise noted.

### 3D Collagen I Gel Culture

Three-dimensional collagen I gels were prepared by mixing cells with the following reagents: DMEM (14%), FBS (10%), 5× Conc. DMEM (16%), 0.1N NaOH (10%) and 4mg/mL collagen I (50%) (MP Biomedicals). The final collagen concentration is 2mg/mL within each construct. Constructs of a volume of 1.0mL were made in 12-well plates and the cellular density was kept at 1.0×106 cells per mL ECM. The constructs were incubated at 37

C for 30 minutes, released from the wells and incubated in DMEM for 24 hours.

### Fluorescence Imaging

Constructs were washed with PBS (phosphate saline buffer Ph 7.4) followed by 30 minute incubation at 4

C in 3% paraformaldehyde. The constructs were then washed with PBS and incubated at 4

C for 30 minutes in cell blocking solution (0.25% Tween20, 1% Bovine Serum Albumin in PBS). Collagen constructs were incubated for 45 minutes in a 1∶500 dilution of phalloidin (Invitrogen) and 10 minutes at room temperature in SYTOX Green Dye (Invitrogen) at a final concentration of 50nM in cell blocking solution. The samples were stored at 4

C in PBS until imaging. 3D confocal images were taken using a Zeiss LSM 51 and ImageJ (NIH) was used to convert the files into.tiff format for segmentation.

### Second Harmonic Generation (SHG)

Collagen I constructs were harvested at days 0, 0.5, 1, 2 and 3 and second harmonic micrographs were obtained using a Zeiss LSM 510 two photon confocal microscope (Zeiss Inc., Thornwood, NY). 820nm light was shown onto the specimen using a two-photon laser and reflected light is collected at about half the wavelength, 480nm. Images were taken at a 40× magnification. Corresponding cellular images were taken as stated above and merged with the SHG images using Image J (NIH, Bethesda, MD).

### Nuclei Detection and Visualization

3D confocal images were segmented using the Otsu Thresholding algorithm [34] using the ITK software [35]. Otsu thresholding algorithm assumes there are two classes of pixels in the observed image and finds a threshold value 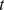 that will automatically separate the foreground pixels from the background pixels. The algorithm uses the zeroth and first order statistics of the input image histogram to find the optimum threshold value. Given 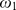, 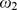 as the probabilities of observing class 1 and 2 (background and foreground) and 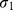 and 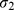 as the corresponding variances of the intensities values in class 1, class 2 respectively, Otsu algorithm searches for the threshold value that minimizes the intra-class variance defined as in equation (1),
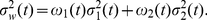
(1)


Otsu exhaustively searches a threshold value that minimizes the intra-class variance 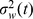.

After finding the optimum threshold value, intensity values of each pixel were compared against the threshold and pixels with intensity values higher than the threshold value were assigned as foreground pixels. The connected foreground pixels were found and the center of mass of these nuclei was calculated and the coordinates of the center were stored. The result of the segmentation was visualized in Figure 2 using the Visualization Toolkit VTK [36].

### Cell-Graph Formation

After identifying the cells in a tissue, a graph was embedded to capture the spatial and structural properties of the tissue. Formally, a graph is represented by 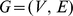 where 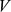 is the vertex set and 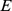 is the edge set of the graph. In our construction, each cell in a tissue constituted a vertex in cell-graphs. An edge 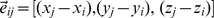 where 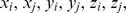 are the coordinates of the 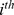 and 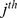 nodes, was introduced if nodes 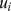 and 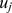 are “close” to each other. In other words, a communication was hypothesized by setting a link between two nodes if the euclidean distance between them was less than a threshold. The Euclidean distance between two cells was simply given by equation (2)
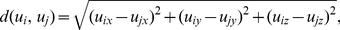
(2)where 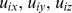 refer to x, y, and z coordinates of node 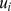, respectively.

The link threshold, gives the maximum allowed distance for two cells to communicate with each other. The higher this edge threshold is, the more cell-graph edges are introduced in the construction. The edge lengths cannot be smaller than the length of the shorter axis of cell membrane. We performed a parametric search and repeated our experiments within the range of 10 to 180nm to find that a specific edge length between 55, 60nm gives the observed faster maturation.

### Computing Collagen Alignment Index

This study develops a new methodology to calculate the Collagen Alignment Index (CAI) to quantify the 3D spatial organization of MSC and its type I collagen microenvironment during differentiation and remodeling. The algorithm describing the steps for computing CAI is shown in algorithm 7 at the end of the paper.

In order to model cooperative pulling between a pair of cells and alignment of the collagen fibers, we partitioned the 3D space into disjoint compartments using Voronoi diagrams. The Voronoi partitioning ensures that each cell-graph edge resides in a unique compartment as depicted in Figure 3(b). The midpoint of each cell-graph edge was input as a “seed point” to Voronoi diagram construction which partitioned the tissue space into convex polygons such that each polygon contained exactly one seed point and every other point in a given polygon was closer to its seed point than to any another seed point in the tissue. Each such polygon constitutes a Voronoi cell in 3D.

For every pixel in a given Voronoi cell, the gradient vector and its magnitude were calculated. The gradient vector of a pixel points in the direction of the greatest rate of change in the intensity and the magnitude is the amount of the change. The distribution of angles between each gradient vector and the cell-graph edge was calculated. The angle 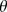 between two vectors 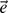 and 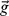 is given by
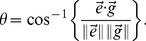
(3)


The angle 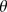 describes the direction of the primary type I collagen organization found within that space with respect to the hypothesized cell communication link as depicted in Figure 4.

The distribution of 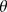 values weighted by the magnitude of the gradient vectors was drawn and a histogram was formed. This histogram was then normalized by the total gradient magnitude. This normalization ensured that the histogram was a probability density function. That is, given an angle, the histogram gave the probability of observing that angle with respect to the communication link between cells. This distribution of 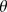 provides information as to the global alignment and organization of the collagen fibrils with respect to the nearest cells. For example a perpendicular gradient vector to an image edge (i.e., collagen fibril) indicates a perfect linear alignment and the distribution has a high peak at 90.

### Computing Cell Dissatisfaction Level

A cell-graph edge 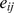 is incident to two nodes, 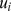 and 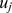. After projecting the collagen forces onto cell-graph edges, each edge was under two different forces: one exerted by node 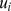 and another one by node 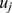. These two force vectors are acting along the same cell-graph edge. For each cell-graph edge, the vectoral sum of these two forces were found and its magnitude was assigned to the edge as the edge's weight. This weight was referred as the dissatisfaction of the collagen alignment from a local perspective. From a global perspective, the total dissatisfaction of the tissue was found by summing each weight dissatisfaction values of the edges.
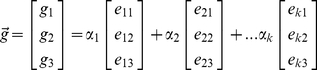
(4)

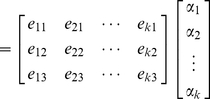
(5)

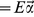
(6)


The net pull of ECM by each node was projected onto cell-graph edges. Note that these forces are not necessarily parallel to any of the edge vectors. Moreover, for a given node 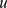, the cell-graph edges incident to 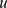 do not necessarily define an orthonormal space. That is, the cell-graph edges are not unit length and they are not perpendicular to each other. This complicates the projection of the net pull force. The net gradient vector of a cell can be written as a linear combination of the cell-graph edge vectors emanating from node 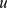 as in equation (4) where 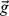 is the net-force vector at node 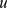 and 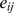 is the 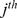 component of the 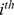 edge. Since there is possibly more than 3 cell graph edges, this linear system is underdefined and have infinitely many solutions in most cases. To overcome this problem, we obtained the minimum norm solution that is addressed at the end of this section (equation (7)) when the system is underdefined.

Equation (4) can be rewritten in a more compact form as in (5). The solution of this system assigns each cell-graph edge a weight given by 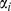. This quantitative value was assigned as the *Cell Dissatisfaction Level* (CDL) corresponding to that edge and quantifies the dynamic remodeling ‘forces’ exerted by individual cells on their local microenvironment. Tissue satisfaction is reached upon homeostasis when the forces exerted by cells in a local area are balanced. The Global Dissatisfaction Level (GDL) of the tissue was found by summing the weights projected onto each cell graph edge, and presented in Figure 6. MSC in type I collagen gels slowly progressed from a dissatisfied state towards a more satisfied state (decrease in dissatisfaction) over time as fibrillogenesis and reorganization occurs. When treated to differentiate, MSC accelerated their progression towards a more ‘satisfied’ state, Figure 6. By filtering out the weights of the cell-graphs that are less than a certain threshold, local microenvironment where fibrillogenesis and reorganization has reached a certain degree can be identified and visualized as depicted in last row of Figure 2.

### Projections

The gradient vectors were summed to find the *net pull* direction that has the most intensity change in a compartment. For each cell, the net pull vector was written as a linear combination of the cell-graph edge vectors as in equation (4) where 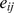 is the 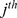 component of the 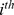 edge. The solution to equation (6) was unique only if node 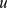 has exactly three edges (i.e., the degree of node is exactly 3) that did not constitute a plane. If the degree of node was not equal to 3 then special cases were addressed as discussed below.

#### Less than 3 Edges

In the case of a node having less than three edges (ie a node with 1 outgoing edge or a node with 2 outgoing edges), the net gradient vector cannot be represented as a summation of projections onto the cell-graph edges. In this case, a hypothetical edge, 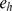 is introduced to make the system solvable. When there is only 1 edge missing, a new edge that is not in the same plane as the other two is introduced. The newly introduced edge is the cross product of the existing two cell-graph edges, that is 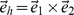. After the introduction of the third edge the system is solved by 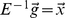.

When a given node has only one edge 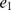, a similar methodology is performed as in the previous case. Two hypothetical edges are introduced to make the system solvable. The first edge is chosen such that it is perpendicular to the existing edge 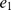. The choice of this perpendicular line is arbitrary as there are infinitely many perpendicular lines to 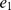. After introducing this new edge 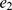, total number of edges incident to this node is now two and only one edge is missing. To make the system solvable the cross product is obtained and assigned as the third edge, 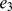, as in the previous case. All these edges are perpendicular to each other and therefore the system is solvable now. The solution vector 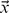 is obtained by inverting the matrix 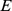 and multiplying it with 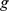 as in the previous case.

#### More than 3 Edges

Another special case occurs when a node 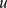 has more than 3 edges incident to that node. The equation system in (6) becomes underdefined. That is, the net pull exerted by the collagen can be projected to the cell-graph edges in infinitely many ways. This is because 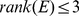 no matter how many more edges 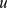 has. There are two possible ways to overcome this issue: either restrict the number of edges a node can obtain or fix one of the infinitely many solutions by some constraints. A common constraint is to use the solution vector 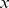 with the minimum norm. The most commonly used vector norm is the 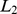-norm which is defined as 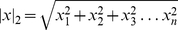. The solution vector with the minimum norm is referred to as the minimum norm solution and can be obtained by
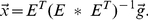
(7)


### Statistical Analysis

We used the Kolmogorov-Smirnov test (KS test) to verify our observation that the pdfs of the 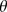 values for the treated group at hour 12 and the untreated group at day 2 were coming from the same distribution (see Figure 5). KS-test calculates the empirical cumulative distribution functions, ECDFs, for each data set. ECDF for a given a dataset 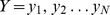 is defined as 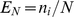 where 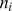 is the number of points less than 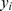 and 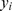 are ordered from smallest to largest value. The maximum difference of two ECDFs is zero when the supplied samples are coming from the exact same distribution.

We used the *kstest2* function of the Matlab software with a confidence interval of %5. Only the histogram values in [60,120] range are used.
